# Evaluation of Parkinson’s Disease Motor Symptoms via Wearable Inertial Measurements Units and Surface Electromyography Sensors

**DOI:** 10.3390/bioengineering12101116

**Published:** 2025-10-18

**Authors:** Xiangliang Zhang, Wenhao Pan, Zhuoneng Wu, Xiangzhi Liu, Yiping Sun, Bingfei Fan, Miao Cai, Tong Li, Tao Liu

**Affiliations:** 1 The State Key Laboratory of Fluid Power and Mechatronic Systems, School of Mechanical Engineering, Zhejiang University, Hangzhou 310027, China; xlzh@zju.edu.cn (X.Z.); 3220102524@zju.edu.cn (Z.W.); liuxiangzhi@zju.edu.cn (X.L.); liutao@zju.edu.cn (T.L.); 2 The Key Laboratory of Education, Ministry for Modern Design and Rotor-Bearing System, Xi’an Jiaotong University, Xi’an 710049, China; panwh@stu.xjtu.edu.cn; 3 The Second School of Clinical Medicine, Zhejiang Chinese Medical University, Hangzhou 310053, China; sunyiping@zcmu.edu.cn; 4 The College of Mechanical Engineering, Zhejiang University of Technology, Hangzhou 310014, China; 5 The Department of Neurology, Zhejiang Hospital, Hangzhou 310030, China; 6 The Department of Sports Science, College of Education, Zhejiang University, Hangzhou 310027, China

**Keywords:** Parkinson’s disease, movement disorder, gait, surface electromyography, Unified Parkinson’s Disease Rating Scale

## Abstract

Parkinson’s disease (PD) is one of the fastest-growing neurodegenerative disorders; its cardinal motor signs—tremor, bradykinesia, and rigidity—substantially impair quality of life. Conventional clinician-rated scales can be subjective and exhibit limited interrater reliability, underscoring the need for objective and reliable quantification. We present an integrated evaluation framework that leverages surface electromyography (sEMG) with multimodal sensing. For representation learning, we combine time–frequency descriptors with Mini-ROCKET features. Grading is performed by an sEMG-based Unified Parkinson’s Disease Rating Scale (UPDRS) model (LDA-SV) that produces per-segment probabilities for ordinal scores (0–3) and aggregates them via soft voting to assign item-level ratings. Participants completed a standardized protocol spanning gait, seated rest, and upper-limb tasks (forearm pronation–supination, finger-to-nose, fist clench, and thumb–index pinch). Using the aforementioned dataset, we report task-wise performance with 95% confidence intervals and compare the proposed model against CNN, LSTM, and InceptionTime using McNemar tests and log-odds ratios. The results indicate that the proposed model outperforms the three baseline models overall. These findings demonstrate the effectiveness and feasibility of the proposed approach, suggesting a viable pathway for the objective quantification of PD motor symptoms and facilitating broader clinical adoption of sEMG in diagnosis and treatment.

## 1. Introduction

Parkinson’s disease (PD) is among the most rapidly increasing neurological disorders worldwide, with an overall prevalence of approximately 1.51‰ [1] and an estimated 1.86% among individuals aged ≥65 years in China [2]. Core motor manifestations—tremor, bradykinesia, rigidity, and gait disturbance—substantially reduce patients’ independence [3]. Marked disease heterogeneity across phenotypes and disease stages further complicates clinical assessment and individualized management [4]. The Movement Disorder Society–Unified Parkinson’s Disease Rating Scale (MDS-UPDRS) remains the standard for quantitative evaluation; Part III of UPDRS (UPDRS-III) systematically examines upper-limb movements, gait, posture, and tremor [5]. However, ratings depend on clinician expertise and the examination context, leading to subjectivity and limited inter-center consistency, which underscores the need for objective and reproducible assessment techniques.

Surface electromyography (sEMG) offers a noninvasive readout of muscle activation and is more suitable for long-duration wearable monitoring than needle EMG. sEMG arises from the superposition of motor-unit action potentials propagating along muscle fibers and filtered by subcutaneous tissues at the skin surface [6]. It has been used to identify a range of upper-limb actions, as well as rest states [7,8]. In practice, sEMG is vulnerable to electronic noise, skin conditions, electrode displacement, and inter-individual variability [9]. In parallel, inertial measurement units (IMUs)—integrating accelerometers and gyroscopes, and in some devices, magnetometers—provide tri-axial acceleration and angular velocity signals (with optional magnetic field), enabling high signal-to-noise ratio (SNR) and stable measurements of segment motion and orientation. Prior studies have utilized wearable IMUs to estimate plantar forces [10], infer thigh posture from shank-mounted sensors [11], and recognize and quantify PD gait impairments [12]. Because sEMG (muscle activation) and IMU (kinematics) convey complementary information, multimodal fusion can improve phase delineation, event detection, and symptom quantification with greater robustness and interpretability.

We therefore adopt a multimodal framework that jointly acquires sEMG and IMU signals and couples them with advanced time-series modeling to enable objective assessment of PD motor symptoms. We first compute handcrafted descriptors and Mini-ROCKET–derived features and then develop an sEMG-feature–based UPDRS scoring model (LDA-SV). The remainder of the paper is organized as follows: Section 2 reviews related work; Section 3 details the experimental protocol, data preprocessing, feature-extraction procedures, and the LDA-SV model; Section 4 reports the empirical results; Section 5 discusses the findings; and Section 6 concludes the study and outlines future directions.

## 2. Related Works

Research on the objective assessment of PD motor symptoms has recently focused on four task domains—rest tremor, rigidity, upper-limb movements, and gait—with an emphasis on how task design, sensing modality, and sensor placement impact data quality and discriminative performance. To improve the reliability of sEMG during rest, Zhang et al. proposed a seated posture with the forearm relaxed on an armrest, which yielded more stable signals than the traditional outstretched-arm posture [13]. Nisticò et al. compared sEMG features across armrest, supine, and standing positions in tremor-dominant PD versus essential tremor, suggesting that antagonist co-contraction patterns may aid differential diagnosis [14].

Upper-limb assessment typically relies on standardized tasks (e.g., finger tapping, finger-to-nose, elbow flexion–extension, wrist movements) with synchronized acquisition. Using a wrist-worn sEMG band in 45 patients, Kleinholdermann et al. extracted time/frequency features from channels over superficial agonists and applied multiple regression models to predict UPDRS-III scores [15]. Adem et al. analyzed sEMG data collected during multiple upper-limb tasks, selected entropy and mean-frequency features, and used SVMs to classify disease with an accuracy of over 90% [16]. In a 24-h monitoring study of 16 advanced cases, Rissanen et al. combined sEMG and accelerometry, demonstrating strong correlations with UPDRS-III and identifying periods of abnormal neuromuscular activity consistent with patient diaries [17].

Gait impairment substantially limits mobility in PD, and freezing of gait (FOG)—common in advanced stages—is closely linked to fall risk [18]. Two research threads contrast muscle-activation patterns between individuals with PD and healthy controls; another targets the detection and prevention of FOG. Segmenting gait cycles from accelerometry, Fricke et al. extracted sEMG time–frequency features, reduced dimensionality, and found SVMs performed best for differentiating multiple abnormal gait phenotypes [19]. Using wavelet/time–frequency analyses, Nardo and Romanato et al. reported a shift toward simplified muscle-recruitment strategies in PD [20]; Romanato et al. further observed delayed and abnormal distal-muscle activation at key gait phases and altered proximal-muscle activity (stronger during load acceptance, weaker during swing) [21]. In multimodal FOG detection (EEG, sEMG, acceleration, and electrodermal activity), Zhang et al. showed that EEG is highly predictive of FOG, while long-term follow-up remains more practical with wearable sEMG/IMU systems [22]. Moore et al. developed a wearable sEMG device for real-time lower-limb monitoring and preemptive FOG alerts to reduce falls [23].

For rigidity, LIU et al. analyzed sEMG during passive upper-limb motion in 51 patients, showing that integrated EMG correlates positively with rigidity level and offers a pathway to quantitative assessment [24]. Meigal et al. collected sEMG during voluntary isometric contraction and during spontaneous normal tone, examining multifeature relations to rigidity [25]. For rest tremor, efforts include optimizing posture/task protocols to improve sEMG quality [13,14] and fusing sEMG with IMU/accelerometry to characterize tremor statistics and time-varying properties using time–frequency and wavelet techniques [26].

Handcrafted sEMG features span time, frequency, and time–frequency domains; for example, Kleinholdermann et al. used seven such features to predict upper-limb UPDRS items [15]. Cross-channel descriptors—such as co-contraction indices [21,27] and antagonist phase differences [14,28]—capture inter-muscle coordination. On the representation-learning front, Rezaee et al. extracted high-level features via pretrained CNNs (AlexNet, CaffeNet, and VGG-F) followed by dimensionality reduction [29]. Yin et al. combined autoencoder feature fusion with the MSC-TimesNet model to improve classification accuracy [30]. Meanwhile, time-series classification (TSC) has advanced rapidly: HIVE-COTE integrates cross-domain representations and multiple classifiers [31,32]; CIF samples random intervals to derive discriminative features paired with random forests [33]; the ROCKET family uses random convolutional kernels for fast multi-scale mapping [34,35]; and InceptionTime adapts Inception-style multi-scale convolutions for complex temporal patterns [36]. These methods have been validated on the UCR [37] and UEA [38] datasets and provide guidance for joint sEMG/IMU modeling.

sEMG-based quantification shows promise for early screening, treatment evaluation, and home monitoring in PD. Moghadam et al. distinguished early PD from healthy controls using hand-tremor sEMG in 33 cases [39]. As therapies evolve, the demand for objective outcome measures has grown, making wearables increasingly central [40]. For example, Mittal et al. analyzed rhythmic burst activity to identify tremor-active forearm muscles and compared muscle function before/after botulinum-toxin injections [41]; Chang et al. examined how whole-body vibration training modulates lower-limb activation, informing rehabilitation strategies [27]. Overall, the multimodal fusion of sEMG and IMU, paired with appropriate task paradigms and efficient sequence modeling, is well-positioned to enable continuous, objective, and interpretable tracking of PD motor symptoms in both clinical and home settings.

## 3. Materials and Methods

### 3.1. Participants and Protocols

This study enrolled two cohorts: a PD group with UPDRS Part III item-level ratings restricted to 0–3 (i.e., no item scored 4) and a healthy control group with all items equal to 0. The inclusion criteria for the PD cohort were a confirmed PD diagnosis, the ability to perform upper-limb tasks, and the ability to ambulate independently without the assistance of caregivers or assistive devices. Healthy controls had no history of gait impairment. The protocol was approved by the Ethics Committee of the Zhejiang University School of Medicine (No. 2021-39), and written informed consent was obtained from all participants.

The PD cohort comprised 17 individuals. Each participant’s UPDRS was independently scored by two clinicians; inter-rater reliability was evaluated using ICC(2,1) and weighted kappa, and any discrepancy was adjudicated by a third clinician, whose decision served as the final score. The cohort included 14 males and 3 females (age 71.0±8.2 years; height 1.66±0.12 m; body mass 62±10 kg). Subtype distribution was tremor-dominant (TD, n=10) and rigidity-dominant (RD, n=7); Hoehn and Yahr stages were I (n=7), II (n=4), and III (n=6). The healthy control cohort included 11 young adults without any history of gait impairment (9 males, 2 females; age 26.3±6.4 years; height 1.71±0.15 m; body mass 65±8 kg).

**Figure 1:**
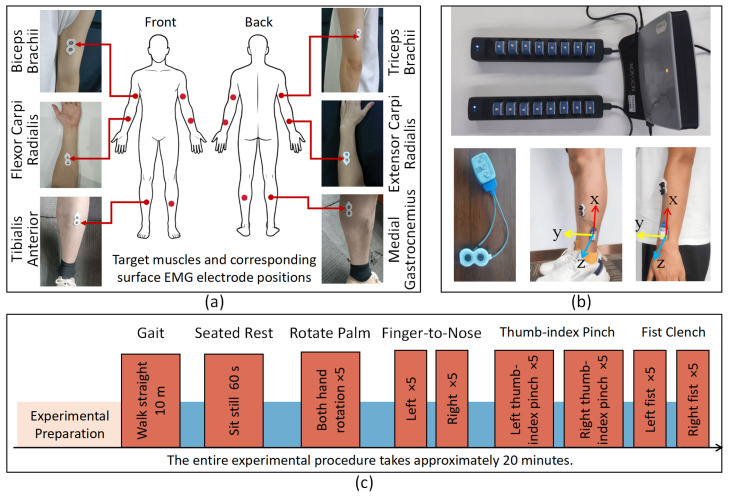
(**a**) Target muscles and corresponding surface EMG electrode position. (**b**) Experimental equipment and IMU placement. (**c**) Experimental procedure and tasks.

We used the Noraxon Ultium EMG system (Noraxon USA, Scottsdale, AZ, USA) or synchronous acquisition of surface EMG (sEMG) and inertial signals. The sEMG sampling rate was 2000 Hz, and the IMU sampling rate was 200 Hz. In total, 12 wireless sensors were deployed: eight sEMG-only units and four dual-mode units that recorded sEMG and a 9-axis IMU (accelerometer, gyroscope, and magnetometer) concurrently, enabling strict time alignment between myoelectric and kinematic data. sEMG electrodes were bilaterally positioned over the biceps brachii, triceps brachii, flexor carpi radialis, extensor carpi radialis, tibialis anterior, and medial gastrocnemius, as shown in Figure 1a. The four dual-mode modules were mounted on the lateral shanks (2–5 cm proximal to the lateral malleoli) and the lateral forearms (1–3 cm proximal to the wrists; one per limb); their electrode leads were routed to the target muscles so that both sEMG and segment kinematics were captured simultaneously, as shown in Figure 1b.

Three sub-experiments were conducted: gait, seated rest, and upper-limb movements. In the gait experiment, each participant completed three independent 10 m walking trials. During seated rest, participants sat upright in an armchair, relaxed their upper limbs, and maintained the posture for 60 s. The upper-limb protocol included forearm pronation–supination, a finger-to-nose task, and simple motor tasks (fist clench and thumb–index finger pinch). In the pronation–supination task, participants raised both arms in front of the chest to a horizontal position (parallel to the floor) and, while maintaining this posture, simultaneously alternated between pronation and supination for seven consecutive cycles before returning to the start position. In the finger-to-nose task, participants maintained the same seated posture, touched the nasal tip with the index finger of one hand, and returned to the start position while keeping the contralateral upper limb still, performing five repetitions per side. In the simple motor tasks, participants performed a fist clench and a thumb–index finger pinch with one hand at a time while the opposite upper limb remained stationary, five trials per side, as shown in Figure 1c.

### 3.2. Feature Extraction Methods for Multichannel Surface EMG Signals

#### 3.2.1. Data Preprocessing

To derive cycle-aligned features from raw surface EMG, we leveraged simultaneously recorded inertial signals to identify and segment movement cycles precisely. IMU data were first denoised using wavelet soft-thresholding. Cycles for the forearm pronation–supination and finger-to-nose tasks were detected by matching alternating zero crossings and peaks from the *z*-axis angular velocity of the wrist-mounted IMUs. Toe-off and heel-strike events were identified from the *z*-axis angular velocity of the shank-mounted IMUs, enabling delineation of gait cycles [42]. Because the seated rest and rigidity examinations lack stable periodicity, these continuous signals were partitioned into equal-length segments.

For sEMG preprocessing, a digital filter cascade was applied: a 500 Hz low-pass filter (LPF) to suppress high-frequency baseline noise while retaining the informative sEMG band, a 10 Hz high-pass filter (HPF) to remove low-frequency artifacts, and notch filters (NF) at 50 Hz and its harmonics (100, 150, 200 Hz, etc.) to eliminate power-line interference and harmonic contamination.

#### 3.2.2. Feature Extraction Methods

To capture both local dynamics and global trends in sEMG during feature extraction, we adopted a sliding-window segmentation strategy with a fixed window length of 200 ms and 50% overlap. Within each window, time- and frequency-domain descriptors were computed independently, converting each single time series into a set of window-level features. For a given examination, let *l* denote the number of windows obtainable from one motion cycle (or one equal-length segment), *m* the number of valid sEMG channels, and *n* the number of features per window. The feature representation for each cycle/segment thus forms an l×m×n tensor. We then applied feature-wise min–max normalization across channels so that each descriptor was scaled with the same linear factors across channels, preserving inter-channel contrasts for the same feature while removing unit and range discrepancies across different features.

We further employed the Mini-ROCKET algorithm [35] to derive multiscale, cross-channel features from sEMG segments. The pipeline converts time-series inputs into a fixed-length feature vector in five stages:

(1) Base kernel design.All convolutional kernels are generated from a set of fixed-weight base kernels. Each kernel has length 9 to capture the smallest local temporal variations; the weights are −1 and 3, yielding 84 base kernels in total. Every kernel is slid along the time series to compute convolution outputs that feed subsequent steps.

(2) Kernel dilation. To encode patterns at multiple temporal scales, Mini-ROCKET applies dilations of the form $2^{n}$, with the maximum dilation determined jointly by the target feature count and the series length. Small dilations emphasize short-range dynamics, whereas large dilations capture long-range structure. To balance scales, approximately comparable numbers of features are allocated to each dilation level.

(3) Bias allocation. Here “bias” differs from the conventional neural-network bias and is introduced to enhance robustness. For each kernel, a sample series is convolved to obtain an output vector. A low-discrepancy sequence is used to generate quantile levels; the corresponding quantiles of the convolution output are taken as bias values. Multiple biases per kernel allow a single kernel to yield multiple features.

(4) Proportion of Positive Values (PPV) pooling. For a convolution output vector *C* and a given bias, the PPV is defined as

(1) $$\mathrm{PPV}=\frac{\#\{c\in C:\;c>\mathrm{bias}\}}{\left|C\right|}$$

mapping each kernel-bias pair to a scalar feature in [0,1].

(5) Feature vector assembly. PPVs are concatenated in a fixed order across kernels, dilations, and biases—optionally per channel and then aggregated—to produce a deterministic, fixed-length feature vector for each sample.

### 3.3. UPDRS Score Prediction Model Based on sEMG Features

**Figure 2:**
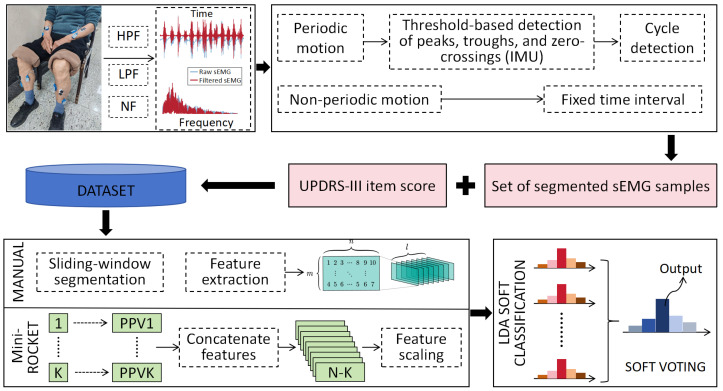
Overall framework of the proposed model.

We propose an sEMG-feature-based UPDRS scoring model (LDA-SV) comprising two stages. First, Linear Discriminant Analysis (LDA) is used to build a soft classifier and generate a probability distribution over the 0–3 ordinal scores. Second, a soft-voting aggregation integrates the segment-level probabilities to determine the participant’s most likely score for the corresponding item, as shown in Figure 2.

We used group-stratified 5-fold cross-validation. For the LDA-SV classifier, we applied class-balanced priors and sample weights to counter class-frequency disparity in each training fold. Specifically, with total training samples *N*, number of classes *K*, and class-*c* count $N_{c}$, we set

(2) $$w_{c}=\frac{N}{K\;N_{c}}.$$

We passed $w_{c}$ as sample weight when fitting the classifier and set class priors proportional to $w_{c}$. Mini-ROCKET feature extraction remained unchanged; no over/under-sampling was performed.

Based on the obtained feature representations, we adopt Linear Discriminant Analysis (LDA) to learn a linear mapping that embeds the observations into a lower-dimensional subspace. The criterion enlarges the dispersion among different class centers relative to the variability inside each class so that samples from the same category form compact clusters, whereas samples from distinct categories are well separated.

Using the extracted features, LDA is further employed as a linear projection tool into a reduced space. Its objective is to maximize the quotient of inter-class scatter to intra-class scatter, encouraging within-class compactness and between-class separation to the greatest extent.

Suppose the dataset contains *K* classes. Denote the mean of class *k* by $\mu_{k}$ and the global mean by μ. The inter-class (between-class) scatter matrix $\Sigma_{B}$ and the intra-class (within-class) scatter matrix $\Sigma_{W}$ are defined as

(3) $$\left\{\begin{array}{cc} \Sigma_{B} & =\sum_{k=1}^{K}n_{k}\;\left(\mu_{k}-\mu \right){\left(\mu_{k}-\mu \right)}^{\;⊤}\\ \Sigma_{W} & =\sum_{k=1}^{K}\;\sum_{x\in C_{k}}\left(x-\mu_{k}\right){\left(x-\mu_{k}\right)}^{\;⊤} \end{array}\right.$$

where $n_{k}$ is the number of samples in the *k*-th class and $C_{k}$ denotes its sample set. The goal is to find a linear transform *a* that reduces the intra-class spread while increasing the inter-class spread, which can be written as the maximization of the following Rayleigh quotient:

(4) $$a=\arg\max_{a}\frac{a^{⊤}\Sigma_{B}a}{a^{⊤}\Sigma_{W}a}$$

This optimization problem can be transformed into a generalized eigenvalue problem using the Lagrangian multiplier method:

(5) $$S_{B}w=\lambda S_{W}w$$

The eigenvector corresponding to the largest eigenvalue represents the optimal linear transformation *w*. Subsequently, the high-dimensional sample features are projected onto the low-dimensional space defined by *w*, where classification is performed based on the distances from the samples to the class centers in this space.

To enhance robustness, we adopt a soft-labeling scheme that fuses UPDRS estimates from multiple motion segments. Instead of returning a single class index, the method outputs posterior probabilities for all classes. For *K* categories, the result is the vector $\pi ={\left(\pi_{1},\ldots ,\pi_{K}\right)}^{⊤}$ with $\pi_{k}=\mathrm{Pr}\left(Y=k\;|\;z\right)$ for an input feature vector *z*.

Assume the class-conditional distribution of *z* is multivariate normal with a shared covariance:

(6) $$p\left(z|Y=k\right)=N\;\left(z;\;\mu_{k},\Sigma \right)$$

Its density can be written explicitly as

(7) $$p\left(z|k\right)=\frac{1}{{\left(2\pi \right)}^{m/2}\;{\left|\Sigma \right|}^{1/2}}\exp\;\left[-\frac{1}{2}{\left(z-\mu_{k}\right)}^{⊤}\Sigma^{-1}\left(z-\mu_{k}\right)\right]$$

where *m* is the feature dimension. By Bayes’ rule, the posterior probability for class *k* is

(8) $$\mathrm{Pr}\left(Y=k|z\right)=\frac{\;p\left(z|k\right)\;\pi_{k}\;}{\sum_{i=1}^{K}p\left(z|i\right)\;\pi_{i}}$$

with $\pi_{k}$ denoting the class prior. Using empirical priors $\pi_{k}\approx N_{k}/N$ from the training data (where $N_{k}$ and *N* are the class and total sample counts), we obtain

(9) $$\mathrm{Pr}\left(Y=k|z\right)=\frac{\frac{N_{k}}{N}\;\exp\;\left[-\frac{1}{2}{\left(z-\mu_{k}\right)}^{⊤}\Sigma^{-1}\left(z-\mu_{k}\right)\right]}{\sum_{i=1}^{K}\frac{N_{i}}{N}\;\exp\;\left[-\frac{1}{2}{\left(z-\mu_{i}\right)}^{⊤}\Sigma^{-1}\left(z-\mu_{i}\right)\right]}$$

Therefore, the soft-classification output for *z* is $\pi ={\left(\mathrm{Pr}(Y=1|z),\ldots ,\mathrm{Pr}(Y=K|z)\right)}^{⊤}$.

Using soft classification, we estimate for each segment a probability distribution over the UPDRS ordinal scores (0–3); the segment-level posteriors are then aggregated via soft voting to determine the participant’s most likely score for the corresponding item.

Soft voting is an ensemble learning strategy used to combine multiple soft-classification outputs for the same sample.Within one motor examination, the sEMG recording of a subject is segmented into *n* valid fragments ${\left\{x_{i}\right\}}_{i=1}^{n}$. Each fragment is fed to the same classifier to obtain a posterior rating distribution. Let *x* denote the subject-level input and $x_{i}$ the *i*-th fragment. The classifier returns, for each class (rating) *k*, the fragment-wise posterior

(10) P(y=k∣x,i),(i=1,2,…,n)

Because all fragments are considered to contribute equally, soft voting aggregates these posteriors by simple averaging to form a subject-level probability distribution:

(11) $${\bar{P}}_{\Sigma }\left(y=k|x\right)\;=\;\frac{1}{n}\sum_{i=1}^{n}P\left(y=k|x,i\right)$$

The final predicted class (rating) is then chosen as the one with the largest aggregated posterior:

(12) $$\hat{y}\;=\;\arg\max_{k}\;{\bar{P}}_{\Sigma }\left(y=k|x\right)$$

The resulting $\hat{y}$ serves as the subject’s UPDRS-III item score for the corresponding task and is used as the final clinical estimate of motor symptoms.

**Figure 3:**
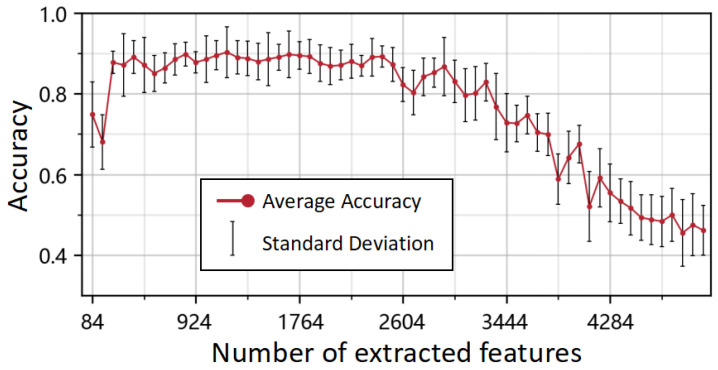
Relationship between accuracy and the number of extracted features.

We performed a grid search to evaluate how the number of Mini-ROCKET features affects classification performance and to select the optimal setting. In Mini-ROCKET, the bank of convolutional kernels is determined by the feature-count hyperparameter; the downstream LDA classifier has no tunable hyperparameters, so only the feature count was adjusted. Because Mini-ROCKET yields a feature count that is always a multiple of 84, we swept from 84 to 5040 in increments of 84. Figure 3 shows the mean and variance of 5-fold cross-validated accuracy as a function of the feature count. When the feature count was 1176, the model achieved the highest mean accuracy (90.31%) for predicting the “Gait” subitem from gait sEMG segments; therefore, all subsequent experiments fixed the feature count at 1176.

## 4. Results

We considered 12 UPDRS-III subitems as prediction targets: Tremor at Rest (TR), Action or Postural Tremor of Hands (AT), Rigidity (RG), Finger Taps (FT), Hand Movements (HM), Rapid Alternating Movements of Hands (RAM), Leg Agility (LA), Arising from Chair (AC), Posture (PO), Gait (GA), Postural Stability (PS), and Body Bradykinesia and Hypokinesia (BBM).

The Accuracy (Equation (13)), Precision (Equation (14)), Recall (Equation (15)), and F1-score (Equation (16)) of the proposed LDA–SV model are summarized in Table 1. Here, TP (true positives) denotes the number of positive samples correctly predicted as positive; FP (false positives) the number of negative samples incorrectly predicted as positive; TN (true negatives) the number of negative samples correctly predicted as negative; and FN (false negatives) the number of positive samples incorrectly predicted as negative.

(13) $$\mathrm{Accuracy}=\frac{TP+TN}{TP+FP+TN+FN}$$

(14) $$\mathrm{Precision}=\frac{TP}{TP+FP}$$

(15) $$\mathrm{Recall}=\frac{TP}{TP+FN}$$

(16) $$F1\mathrm{-score}=\frac{2\times \mathrm{Precision}\times \mathrm{Recall}}{\mathrm{Precision}+\mathrm{Recall}}$$

As comparative baselines, we evaluated three widely used time-series classifiers, convolutional neural network (CNN), long short-term memory (LSTM), and InceptionTime, on the same set of extracted features using an identical preprocessing and cross-validation protocol. Their performance is summarized in Table 1 in terms of Accuracy, Precision, Recall, and F1-score.

As shown in Table 1, the proposed method achieves the highest single-task accuracy on seven of the twelve tasks (AT, FT, HM, LA, AC, and GA) and ties for the best performance on TR, PO, PS, BBM, and RAM—i.e., it reaches the “highest or co-highest” level on 11/12 tasks. The only exception is RG, where InceptionTime outperforms OUR (82.35% vs. 76.47%). Among the leading tasks, the strongest results are observed for LA (97.96%), AC (95.92%), GA (95.92%), AT (96.15%), HM (96.00%), and FT (94.74%). Performance on TR (95.24%), PS (94.74%), and PO (76.47%) is on par with InceptionTime, while RAM (81.25%) and BBM (81.25%) are broadly comparable to CNN/InceptionTime. Precision, Recall, and F1 are generally consistent with Accuracy, indicating no notable metric divergence.

To assess the relative merits of the proposed method against three baselines, we performed pairwise, per-task comparisons between OUR and CNN, LSTM, and InceptionTime. For each task and model pair, we computed the accuracy difference (ΔAcc) on the exact intersection of samples, reported its 95% confidence interval (CI), and used McNemar’s exact test to assess significance. We additionally summarized effect sizes via the cross-fold win rate ($g_{\mathrm{rate}}$) and the log odds ratio (log(OR)), interpreting them jointly with ΔAcc and its CI to avoid bias from any single metric; the results are summarized in Table 2.

## 5. Discussion

This study introduces a Mini-ROCKET–based multimodal feature-extraction scheme coupled with an LDA-SV classifier. From a task-level perspective, the method performs particularly well on gait/posture items (LA, AC, GA, PS) and rhythmic hand movements (AT, HM, FT), suggesting a stronger capacity to model stable, repeatable temporal structures. In contrast, rigidity (RG) favors InceptionTime, likely because this item relies more on multi-scale convolutions to capture fine-grained and non-stationary patterns. Absolute accuracies for RAM, BBM, and PO are modest across models (typically 70–81%), indicating limited separability or class imbalance; targeted time–frequency/dynamical features and data-rebalancing strategies may therefore help widen inter-model gaps and improve robustness.

As shown in Table 2, the most robust gains of OUR over CNN concentrate on LA and AC (positive ΔAcc with significant *p*-values and $g_{\mathrm{rate}}$ near/equal to 100%), indicating consistent and reproducible improvements on these gait/posture tasks. For most other tasks, ΔAcc tends to be positive, but the CIs span zero, and McNemar’s test is non-significant, indicating trend-level advantages. Comparisons against LSTM show a similar pattern (small but mostly non-significant deltas), whereas results versus InceptionTime are largely on par (many ΔAcc≈0 with wide intervals), consistent with the overall picture in Table 1. Note that inflated or unstable log(OR) values often arise when discordant pairs are scarce; these should be interpreted jointly with ΔAcc and its CI. For tasks such as RG, RAM, BBM, and PO, where separability is limited or class imbalance may be influential, future work should incorporate more task-specific time-frequency/dynamical features and rebalancing strategies and improve statistical power and robustness via larger effective samples, stratified cross-validation, exact interval estimation, and multiple-comparison control.

At the group level, the Mini-ROCKET importance profiles reveal a clear time-scale structure. Kernel types with small dilations contribute more to tremor-related tasks (TR, AT), consistent with their higher-frequency, short-latency signatures; medium dilations are more informative for bradykinesia/rigidity and hand-dexterity tasks (FT, HM, RAM, RG, BBM); and large dilations dominate gait/posture tasks (LA, AC, GA, PO, PS), reflecting lower-frequency, multi-cycle control. Features at higher bias quantiles tend to cluster around salient movement phases (e.g., angular-velocity peaks or sEMG bursts). These patterns accord with the task-level performance differences in Table 1 and Table 2 and provide clinically interpretable, time-scale-based cues that help relate model decisions to symptom physiology.

Compared with prior work, our study has several advantages. First, in sensor design, we jointly acquire sEMG and IMU signals, providing complementary information on muscle activity and kinematics. Many earlier studies relied on a single modality; for example, Dai et al. assessed tremor and bradykinesia using only IMUs [43], which lack direct muscle measurements; Rissanen et al. collected both sEMG and accelerometry in home settings [17] but did not further explore cross-modal feature fusion and model optimization; and Romanato et al. incorporated force sensors and force plates to boost accuracy [44], at the cost of higher system complexity and expense, limiting use outside specialized labs. By contrast, our method achieves efficient multimodal fusion while preserving wearability and low cost, making it better suited to applications with real-time constraints.

Our findings also underscore the importance of pairing feature extraction with the correct classifier. Traditional pipelines, e.g., Kleinholdermann et al. using sEMG time–frequency features with SVR to predict UPDRS-III scores [15], can be effective but often struggle to capture complex, nonlinear patterns. Deep learning approaches, such as the stacked-CNN framework proposed by Rezaee et al. [29] and the end-to-end advantages reported by Fricke et al. [19], may improve performance but typically require large datasets and considerable computational resources. Leveraging Mini-ROCKET’s efficient feature mapping with the LDA-SV classifier, our approach exceeds CNN, LSTM, and InceptionTime in accuracy while retaining computational efficiency and interpretability, aligning well with clinical deployment needs.

This study has several limitations. First, the cohort is small (17 patients with PD and 11 controls), and the two groups are substantially age-mismatched, which may confound disease-specific effects and limit generalizability. Second, experiments were conducted under relatively controlled conditions; in real clinical or home environments, sensor displacement, signal noise, and inter-subject variability may degrade performance. Third, although multimodal fusion improved robustness, it also increased the complexity of deployment. In terms of evaluation, we relied on fivefold cross-validation without external or hold-out testing; therefore, the findings should be interpreted with caution. To mitigate overinterpretation, we report task-wise 95% confidence intervals and use McNemar tests with log odds ratios for pairwise method comparisons. Regarding baselines, HIVE-COTE 2.0 was excluded due to its substantial computational overhead and limited alignment with our clinical, per-task evaluation constraints. Instead, we prioritized modern yet tractable comparators (e.g., InceptionTime) under a common preprocessing and validation protocol. Future work will expand and age-match the cohort, add external/hold-out validation, investigate more efficient fusion strategies (including self-supervised and graph models), and evaluate robustness in real-world clinical and home settings.

## 6. Conclusions

This study presented a Mini-ROCKET–based feature mapping pipeline paired with an LDA-SV scoring model to quantify UPDRS-III subitems. Under small-sample 5-fold cross-validation, we report task-wise 95% CIs and McNemar/log(OR) comparisons. Preliminary results indicate that this lightweight framework offers advantages over classic baselines on several hand-dexterity and gait/posture tasks, while a group-level, partially interpretable analysis improves clinical readability. Given the small, age-mismatched cohort and the absence of external validation, findings should be interpreted with caution. Future work will expand and age-match the cohort, add external/hold-out evaluations, and explore more efficient fusion and self-supervised strategies to strengthen generalization and temporal representations.

## Figures and Tables

**Table 1:** Per-task performance of four models.

Task	CNN (%)				LSTM (%)				InceptionTime (%)				OUR (%)			
	Acc	Prec	Rec	F1	Acc	Prec	Rec	F1	Acc	Prec	Rec	F1	Acc	Prec	Rec	F1
TR	90.48	81.95	90.48	85.98	90.48	91.48	90.48	89.15	95.24	96.83	95.24	94.92	95.24	95.50	95.24	94.51
AT	76.92	77.89	76.92	76.92	80.77	77.88	80.77	79.26	88.46	88.63	88.46	88.40	96.15	92.58	96.15	94.30
RG	70.59	69.16	70.59	68.29	76.47	73.28	76.47	74.25	82.35	87.39	82.35	84.71	76.47	71.90	76.47	74.05
FT	84.21	83.68	84.21	82.38	89.47	91.39	89.47	89.25	84.21	86.09	84.21	84.56	94.74	90.06	94.74	92.26
HM	76.00	76.97	76.00	75.88	88.00	88.39	88.00	88.05	92.00	93.33	92.00	92.03	96.00	96.29	96.00	95.41
RAM	81.25	88.28	81.25	81.75	75.00	82.29	75.00	77.37	81.25	87.95	81.25	79.97	81.25	83.33	81.25	81.57
LA	81.63	85.48	81.63	77.84	89.80	90.49	89.80	89.67	95.92	97.96	95.92	96.43	97.96	98.02	97.96	97.93
AC	77.55	84.02	77.55	73.27	85.71	84.52	85.71	84.84	93.88	94.01	93.88	93.88	95.92	96.05	95.92	95.91
PO	70.59	70.38	70.59	70.03	70.59	72.55	70.59	70.87	76.47	76.76	76.47	76.09	76.47	79.46	76.47	74.73
GA	87.76	88.78	87.76	86.72	87.76	87.76	87.76	87.76	93.88	94.90	93.88	93.99	95.92	96.05	95.92	95.84
PS	89.47	91.58	89.47	89.47	84.21	84.80	84.21	84.26	94.74	90.06	94.74	92.26	94.74	95.32	94.74	94.75
BBM	81.25	81.99	81.25	81.25	75.00	77.19	75.00	74.70	81.25	81.56	81.25	81.02	81.25	83.82	81.25	79.05

**Table 2:** Per-task comparison with ΔAcc, McNemar *p*-value and effect sizes.

Compare	Task	ΔAcc			$p_{\mathrm{McNemar}}$	$g_{\mathrm{rate}(\%)}$	log(OR)
		ΔAcc	CI_Low	CI_High			
Our vs. CNN	TR	4.76	−4.52	4.76	1.0000	100.00	3.664
	AT	19.23	−4.24	26.73	0.1250	85.71	0.000
	RG	5.88	−14.32	17.35	1.0000	66.67	2.373
	FT	10.53	−12.88	20.79	0.6250	75.00	0.389
	HM	20.00	−4.41	27.80	0.1250	85.71	−0.053
	RAM	0.00	−12.19	12.19	1.0000	50.00	2.631
	LA	16.33	4.26	16.33	0.0078	100.00	2.660
	AC	18.37	3.92	22.35	0.0117	90.91	1.273
	PO	5.88	−26.02	33.02	1.0000	57.14	−0.100
	GA	8.16	−3.46	12.14	0.2188	83.33	2.045
	PS	5.26	−5.00	5.26	1.0000	100.00	3.555
	BBM	0.00	−28.64	28.64	1.0000	50.00	−0.847
Our vs. LSTM	TR	4.76	−11.59	14.05	1.0000	66.67	0.903
	AT	15.38	−6.52	22.88	0.2188	83.33	0.217
	RG	0.00	−11.47	11.47	1.0000	50.00	2.968
	FT	5.26	−12.81	15.52	1.0000	66.67	0.788
	HM	8.00	−9.79	15.80	0.6250	75.00	0.717
	RAM	6.25	−27.65	35.09	1.0000	57.14	−1.199
	LA	8.16	−3.46	12.14	0.2188	83.33	0.969
	AC	10.20	−2.25	14.18	0.1250	85.71	1.854
	PO	5.88	−20.79	26.31	1.0000	60.00	1.099
	GA	8.16	−3.46	12.14	0.2188	83.33	2.045
	PS	10.53	−7.20	10.53	0.5000	100.00	2.986
	BBM	6.25	−27.65	35.09	1.0000	57.14	−1.199
Our vs. InceptionTime	TR	0.00	0.00	0.00	1.0000	50.00	4.812
	AT	7.69	−9.41	15.19	0.6250	75.00	0.762
	RG	−5.88	−17.35	14.32	1.0000	33.33	2.120
	FT	10.53	−12.88	20.79	0.6250	75.00	0.389
	HM	4.00	−9.74	11.80	1.0000	66.67	1.099
	RAM	0.00	−28.64	28.64	1.0000	50.00	−0.847
	LA	2.04	−1.94	2.04	1.0000	100.00	4.554
	AC	2.04	−7.21	9.13	1.0000	60.00	0.933
	PO	0.00	−20.35	20.35	1.0000	50.00	1.526
	GA	2.04	−7.21	9.13	1.0000	60.00	0.933
	PS	0.00	−10.26	10.26	1.0000	50.00	1.358
	BBM	0.00	−28.64	28.64	1.0000	50.00	−0.847

## Data Availability

The data presented in this study are available on request from the corresponding author due to the ongoing collaborative nature of the project with the hospital.
